# Carbapenem- and colistin-resistant *Enterobacterales* in intensive care unit patients in Mediterranean countries, 2019

**DOI:** 10.3389/fmicb.2024.1370553

**Published:** 2024-04-12

**Authors:** Sandra Dos Santos, Seydina M. Diene, Amina Benouda, Khalid Zerouali, Doaa M. Ghaith, Rasha H. El-Mahdy, Sawsan H. M. El Tayeb, Ilhem Boutiba, Adnene Hammami, Remie Chrabieh, Ziad Daoud, Laurent Mereghetti, Patrice Francois, Nathalie Van Der Mee-Marquet, on behalf of the consortium

**Affiliations:** ^1^Centre d’Appui pour la Prévention des Infections Associées aux Soins Centre Val de Loire, Centre Hospitalier Universitaire, Tours, France; ^2^Faculté de Pharmacie, Aix-Marseille Université, Marseille, France; ^3^Microbes Evolution Phylogeny and Infections (MEPHI), Aix-Marseille Université, Marseille, France; ^4^IHU-Méditerranée Infection, Aix-Marseille Université, Marseille, France; ^5^Laboratoire de Microbiologie, Hôpital Cheikh Zaid, Rabat, Morocco; ^6^Laboratoire de Microbiologie, Centre Hospitalier Universitaire Ibn Rochd, Faculté de Médecine et de Pharmacie, Casablanca, Morocco; ^7^Clinical and Chemical Pathology Department, Faculty of Medicine, Cairo University, Cairo, Egypt; ^8^Department of Medical Microbiology and Immunology, Faculty of Medicine, Mansoura University, Mansoura, Egypt; ^9^Department of Medical Microbiology, Al Hussein University hospitals, Cairo, Egypt; ^10^Laboratoire de Microbiologie, Centre Hospitalier Universitaire Charles Nicolle, Tunis, Tunisia; ^11^Laboratoire de Microbiologie, Centre Hospitalier Universitaire Habib Bourguiba, Sfax, Tunisia; ^12^Department of Dermatology, Lebanese American University Medical Center Rizk Hospital, Beirut, Lebanon; ^13^Department of Biomedical Sciences, Faculty of Medicine and Medical Sciences, Saint George Hospital-UMC, Beirut, Lebanon; ^14^Service de Bactériologie-Virologie-Hygiène, Centre Hospitalier Universitaire, Tours, France; ^15^Genomic Research Laboratory, Infectious Diseases Service, Geneva University Hospitals and Faculty of Medicine, Geneva, Switzerland

**Keywords:** intensive care unit, carriage, carbapenem-resistant *Enterobacterales*, colistin-resistant *Enterobacterales*, Morocco, Tunisia, Lebanon, Egypt

## Abstract

**Introduction:**

The colonization of patients by carbapenemase-producing *Enterobacterales* (CPE) has been associated with heightened mortality, especially in vulnerable individuals within intensive care units (ICUs). Our study aimed to comprehensively assess CPE prevalence among ICU patients across the Mediterranean region pre-COVID-19, conducting a multicenter prevalence study in the first quarter of 2019.

**Methods:**

We collected clinical data and rectal or fecal samples from 256 ICU patients for CPE testing. Additionally, we performed whole-genome sequencing on 40 representative CPE strains to document their molecular characteristics.

**Results:**

Among the 256 patients, CPE was detected in 73 samples (28.5%), with prevalence varying from 3.3 to 69.0% across participating centers. We observed 13 colistin-resistant CPE strains, affecting three ICUs. Genetic analysis revealed highly diverse *E. coli* and *K. pneumoniae* strains, predominantly from international high-risk clones. Notably, *bla*_OXA-48_ and *bla*_NDM-1_ were the most prevalent carbapenemase genes. Molecular typing uncovered potential patient clusters in six centers. Significantly, longer hospital stays were associated with increased CPE carriage (*p* < 0.001). Nine centers across Morocco, Tunisia, Egypt, and Lebanon voluntarily participated.

**Discussion:**

Our study provides CPE prevalence in Mediterranean ICUs and reaffirms established CPE presence in this setting but also provides updates on the molecular diversity of CPE strains. These findings highlight the imperative of reinforcing infection control measures in the participating ICUs to curtail escalated mortality rates, and of strictly applying isolation measures around patients originating from the Mediterranean region when transferred to other healthcare institutions.

## 1 Introduction

The colonization of patients with carbapenemase-producing *Enterobacterales* (CPE) has been associated with increased mortality among vulnerable individuals, particularly those admitted to intensive care units (ICUs) (Dautzenberg et al., 2015; Tischendorf et al., 2016). Preventing the spread of these multidrug-resistant bacteria in ICUs involves a multifaceted approach, including the promotion and strict adherence to hand hygiene rules, proper disinfection of reusable medical equipment, implementation of strict cleaning and disinfection protocols to reduce environmental contamination, the appropriate use of broad-spectrum antibiotics, education and training of healthcare professionals, implementation of surveillance to quickly detect CPE-associated nosocomial infections, and the quick identification and isolation of CPE carriers to prevent transmission to other patients. Complementing the survey of CPE-associated infections and aiding in the implementation of preventive measures to curb the spread of CPE within ICUs, the epidemiological monitoring of patients carrying CPE is a key-tool to document the epidemiology of these bacteria in ICUs. The ongoing epidemiological data facilitates early detection of potential outbreaks, and assists in the better selection of probabilistic treatments to manage locally acquired infections.

Comprehensive epidemiological data on patient CPE-colonization within ICUs in Mediterranean countries are rather limited, in particular prevalence studies. Prior studies predominantly focused on outbreaks associated with *K. pneumoniae* carrying the *bla*_OXA-48_ gene, notably the 2011 Casablanca study (Barguigua et al., 2015), and those in 2014 involving patients from Algeria and Tunisia (Cuzon et al., 2015; Mathlouthi et al., 2016). Another significant source of clonal dissemination has been identified in *K. pneumoniae* carrying the *bla*_NDM-1_ gene, previously documented in Moroccan ICUs in 2011 (Barguigua et al., 2015) and in ICUs in Cairo in 2013 and 2016 (Wassef et al., 2016; Abdulall et al., 2018). A study conducted in 2016 by Hammami and colleagues highlighted that 34.9% of ICU patients at the University Hospital of Tunis harbored CPE with the *bla*_OXA-48_ and/or *bla*_NDM-1_ genes in their intestinal flora (Hammami et al., 2017). Additionally, to the best of our knowledge, the colonization of ICU patients in Mediterranean countries by colistin-resistant CPE has not been extensively reported, except for an outbreak of colistin-resistant *bla*_OXA48_ *K. pneumoniae* in 2015 among ICU patients at the University Hospital of Mahdia in Tunisia (Mansour et al., 2017). In this instance, the resistance to colistin was not attributed to the presence of *mcr* genes within the plasmids carried by this *K. pneumoniae*.

Our study aimed to assess the prevalence of CPE carriage among ICU patients in hospitals across diverse Mediterranean countries. This involved a multicenter prevalence study conducted just before the onset of the COVID-19 pandemic, in the first quarter of 2019 across a group of nine ICUs. Carbapenem-resistant strains, isolated from rectal or fecal samples, underwent molecular analyses to determine their susceptibility to antibiotics. Additionally, whole-genome sequencing was performed on 40 representative CPE strains to document the molecular characteristics of the predominant clones. Examination of the clinical data was also undertaken to investigate the risk factors associated with the intestinal carriage of CPE among patients in the participating ICUs.

## 2 Materials and methods

### 2.1 Study design

A one-day prevalence study was conducted from January 1, 2019, to March 30, 2019, spanning nine centers in hospitals located in Morocco (2 centers), Tunisia (2), Egypt (3), and Lebanon (2). These centers—labeled C1 to C9—participated voluntarily, aiming to enroll 30 consecutive ICU patients in each. Prior consent from patients or their relatives was sought to access medical records and collect fecal or rectal samples for testing CPE.

### 2.2 Data collection

The data were collected by the attending physicians. Collected data encompassed demographic and clinical information, including physical disabilities, significant underlying health conditions, and established risk factors associated with CPE carriage. These risk factors covered the duration of pre-screening hospitalization, prior hospitalization history, and recent antibiotic usage (on the study day and within the preceding 6 months).

## 3 Microbiological study

### 3.1 Clinical samples

Fecal or rectal swabs were collected using swabs with Amies-type preservation medium (Copan Italia SPA, Italy).

### 3.2 CPE detection and characterization

Clinical swabs were promptly screened for third-generation cephalosporin- and carbapenem- resistant *Enterobacterales* using CHROMagar ESBL and mSuperCARBA chromogenic agar plates (CHROMagar, Paris, France) at each participating center. After incubation of the chromogenic agar plates at 37°C for 24 h under aerobic conditions, each colony morphotype was taken into consideration. All *Enterobacterales* strains were then transferred to the central laboratory in Tours, France, following inoculation on a swab with Amies-type preservation medium. In Tours, we controlled the identification of each colony morphotype using Matrix-assisted laser desorption-ionization-time–of-flight mass spectrometry (microflex LT MALDI-TOF, MBT Compass software version 4.2.100.19; Bruker Daltonics, France). Subsequently, all *Enterobacterales* strains underwent antibiotic sensitivity testing using disc diffusion method (EUCAST, 2019), and determination of Ertapenem Minimum Inhibitory Concentration (MIC) (E-test strip; bioMérieux, Marcy-l’Étoile, France) and colistin MIC using a broth microdilution method as advised by EUCAST for *Enterobacterales* (EUCAST, 2019) (colistin UMIC plates; Bruker Daltonics, France). Carbapenemase production was investigated when Ertapenem MIC >0.5 mg/L using the immunochromatographic test RESIST-5 O.K.N.V.I (Coris BioConcept, Belgium) and confirmed using a PCR test for molecular detection of the genes encoding for the five carbapenemases of primary public health concern (*bla*_OXA-48_, *bla*_NDM_, *bla*_KPC_, *bla*_VIM_ and *bla*_IMP_ genes) (Doyle et al., 2012).

### 3.3 Strain selection and sequencing

Among all CPE strains, 40 were thoughtfully chosen after applying the RAPD technique for epidemiological typing, using three primers as previously described (van der Mee-Marquet et al., 2010). To avoid redundancy, strains with similar RAPD profiles from the same center were excluded, retaining only one for sequencing. The chosen strains underwent whole-genome sequencing (WGS) via next-generation sequencers. Genomic DNA, purified using the DNeasy kit (Qiagen), was sequenced on the Illumina HiSeq, generating 100-base pairs (bp) paired-end reads with barcoding using the Nextera XT kit. Data Processing: Read quality assessment was performed using Trimmomatic v0.36, with specified parameters for paired-end reads. Genome assembly was conducted using the SPADES v3.12.0 assembler. Assembled genomes and plasmids^1^ were annotated using the RAST server.^2^ Multilocus sequence typing analysis (*in silico*) developed by Achtamn et al. ^3^was conducted using annotated genomes and submitted to the Center for Genomic Epidemiology database.^4^ The phylogenetic relationships of all isolates were investigated through genomic single-nucleotide polymorphism (SNP)–based analysis (CSIPhylogeny). Additional tools, available at the Center for Genomic Epidemiology,^5^ were used for detecting antibiotic resistance,^6^ mobile genetic elements, ^7^and virulence factors.^8^ The investigation of phylogenetic relationships among isolates involved genomic single-nucleotide polymorphism (SNP)–based analysis (CSIPhylogeny).

### 3.4 Statistical analysis

Statistical analysis was performed using univariable methods, employing chi2 and Fisher’s exact test as appropriate. All tests were 2-tailed, and a significant level of *p* < 0.05 was used.

### 3.5 Confidentiality and ethical aspects

The study was conducted in collaboration with hospital directors, attending physicians, and the infection control pilot team in Tours, France, in adherence to French Healthcare recommendations for infection prevention. Ethical approvals were obtained at eight hospital centers, ensuring anonymization of patient and sample data prior to analysis. Data from center 9 were not included in the study.

## 4 Results

A total of 256 ICU patients were included in the study. The clinical data were available for 226 patients (Table 1). For all but one of the participating centers, the number of patients per center was approximately 30. Due to patient refusals to participate, center C8 enrolled only 16 patients. Out of the 256 patients, 100 were women (44.0%), and 126 were men (46.0%), with ages ranging from 6 days to 102 years (mean age: 57 years). Various medical conditions were observed among the patients, with 15.5% having experienced trauma, 31.4% having diabetes mellitus, and 26.1% having malignancies. Among the patient population, 44.5% had recent hospitalization history, and 48.0% had received antibiotic treatment in the last 6 months. The most common antibiotics used included third-generation cephalosporins (28.1%) and carbapenems (10.1%). On the day of the study, 74.2% of the patients were under antibiotic treatment, which primarily consisted of third-generation cephalosporins (37.2%), carbapenems (20.4%), amoxicillin-clavulanic acid (13.3%), or fluoroquinolones (7.5%).

**Table 1:** Population characteristics according to centers and patient carriage status.

		Number of patients according to											
		Centers									Carriage status		
		C1	C2	C3	C4	C5	C6	C7	C8	C9	Carriers	Non carriers	All patients
Total number of patients		29	31	28	32	29	31	30	16	30	73	183	256
Clinical data													
Age (years)	<1		4	8		21			1		18	16	34
	1–15		4		4	6	1				6	9	15
	16–65	7	19	16	19	1	20	14	5		33	68	101
	66–85	20	4	4	8	1	7	14	8		14	52	66
	>85	2			1						2	3	5
	nk						3	2		30	9	26	35
Sex	Females	11	15	8	12	14	16	17	7	nk	36	64	100
	Males	18	16	20	20	15	15	13	9	nk	37	89	126
Duration of hopitalization before screening (days)	<2	5	3	5	6	26	17	15	2		26	53	79
	2–3	13	5	5	5	2	4	10	2		7	39	46
	4–7	5	7	7	8	1	2	4	3		8	29	37
	>7	5	16	11	13		7	1	9		31	31	62
	nk	1					1			30	10	22	32
Comorbidities before screening	Trauma	5	8	7	9		1	3	2		12	23	35
	Medical	6	15	16	15	29	4	27	13		43	82	125
	Surgical	23	16	12	17		27	3	3		30	71	101
	Immunodepression	1	2	5		23	6	5	1		22	21	43
	Diabetes	10	9	7	11	2	8	19	5		14	57	71
	Cancer	4	3	2			7	4	2		4	18	22
	Hemopathy				1	1		4	1		1	6	7
	Solid tumor	6	4	2	4		11	1	2		8	22	30
Antibiotherapy	since admission	15	27	17	30	5	30	30	13		47	119	167
	3GC^1^	11	16	5	7		30	14	1		22	62	84
	AClav^2^	1	2	5	16			6			6	24	30
	carbapenem	1	14	3	8	3		9	8		16	30	46
	vancomycin	1											
	fluoroquinolone	1	9	5	1			1			3	14	17
Antibiotherapy	since 6 months**	4	3	3	6	3	30	26	6		22	59	81
	3GC^1^	2	3	1	2		22	12			9	33	42
	AClav^2^		1		1	1		5	2		3	7	10
	carbapenem		1	1	2			10	1		3	12	15
	vancomycin										0	0	0
	fluoroquinolone		1		2			3	1		0	7	7
Hospitalization since 6 months		15	14	4	6	2	26	24	10		26	75	101

^1^3GC third-generation cephalosporin, ^2^AClav amoxicillin-clavulanic acid; *C CPE carriers; **out of neonates.

### 4.1 Detection of the CPE strains

A total of 110 strains of carbapenem-resistant *Enterobacterales* were cultured on SuperCarba plates of which 103 (93.6%) were confirmed as CPE strains. The remaining seven strains that did not produce carbapenemase were obtained from four different centers, consisting of two *Proteus mirabilis* strains, one *Morganella morgannii*, one *K. pneumoniae,* and three *E. coli*.

Out of the 103 CPE strains, 61 were *K. pneumoniae* (59.2%), while there were two *K. aerogenes*, 31 *E. coli* (30.1%), seven *C. freundii*, and two *E. cloacae* (Table 2). Among the 31 *E. coli* strains, 20 were resistant to ciprofloxacin (64.5%), 21 to trimethoprim-sulfamethoxazole (67.7%), 12 to gentamicin (38.7%), and 15 to tobramycin (48.4%). Two strains were resistant to all tested aminoglycosides and fluoroquinolones (6.4%) but remained susceptible to fosfomycin, tigecycline, and colistin. Multi-resistance to antibiotics was more prevalent among the 61 *K. pneumoniae* strains. Specifically, 59 were resistant to ciprofloxacin (93.8%), 56 to tobramycin (90.3%), 50 to fosfomycin (83.3%), 45 to gentamicin (75.0%), and 44 to trimethoprim-sulfamethoxazole (73.3%). Twenty-seven strains were resistant to all tested aminoglycosides and fluoroquinolones (43.5%) but remained susceptible to tigecycline (100.0%) and colistin (81.4%). The detected carbapenemase genes included *bla*_OXA-48_ (44.7%), *bla*_OXA-181_ (6.8%), *bla*_OXA-244_ (4.8%), *bla*_NDM-1_ (19.4%), *bla*_NDM-5_ (12.6%), *bla*_NDM-7_ (2.9%), and *bla*_NDM-4_ (1.0%). Ten out of the 103 CPE strains (9.7%) co-carried both *bla*_OXA-48_ and *bla*_NDM_ carbapenemase genes (Table 2). The *bla*_OXA-244_ gene was found to be associated with *E. coli* (*p* = 0.002), and the *bla*_NDM-1_ gene showed an association with *K. pneumoniae* (*p* = 0.011).

**Table 2:** Characteristics of the 103 CPE strains.

	Carbapenemase genes and colistin-resistance according to species and centers									All CPE
	C1	C2	C3	C4	C5	C6	C7	C8	C9	
All CPE	6	8	7	12	26	28	1	4	11	103
*bla*_OXA-48_	6	3	3	2	9	15	1	2	4	45
*bla*_OXA-181_		1	4		1				1	7
*bla*_OXA-244_					5					5
*bla*_NDM-1_		4		4	6	4			1	19
*bla*_NDM-4_					1					1
*bla*_NDM-5_					3	7		1	2	13
*bla*_NDM-7_						1		1	1	3
*bla*_OXA-48 NDM-5_				6	1	1			1	9
*bla*_OXA-48 NDM-1_									1	1
Colistin resistance (*mcr1*)			2 (0)		8 (0)	3 (3)				13 (3)
*K. pneumoniae*	4	3	7	11	16	11	1		8	61
*bla*_OXA-48_	4	1	3	1	6	6	1		4	26
*bla*_OXA-181_			4							4
*bla*_NDM-1_		2		4	6	4			1	17
*bla*_NDM-4_					1					1
*bla*_NDM-5_					2	1			1	4
*bla*_OXA-48 NDM-5_				6	1				1	8
*bla*_OXA-48 NDM-1_									1	1
Colistin resistance (*mcr1*)			2 (0)		8 (0)					10 (0)
*E. coli*		5		1	10	10		3	2	31
*bla*_OXA-48_		2		1	3	4		2		12
*bla*_OXA-181_		1			1				1	3
*bla*_OXA-244_					5					5
*bla*_NDM-1_		2								2
*bla*_NDM-5_					1	4		1	1	7
*bla*_NDM-7_						1				1
*bla*_OXA-48 NDM-5_						1				1
Colistin resistance (*mcr1*)						3 (3)				3 (3)
Other species										
*K. aerogenes*								1	1	2
*bla*_NDM-7_								1	1	2
Colistin resistance										0
*E. cloacae*	2									2
*bla*_OXA-48_	2									2
Colistin resistance										0
*bla*_OXA-48_						5				5
*bla*_NDM-5_						2				2
Colistin resistance										0

### 4.2 Prevalence of CPE strains

**Figure 1:**
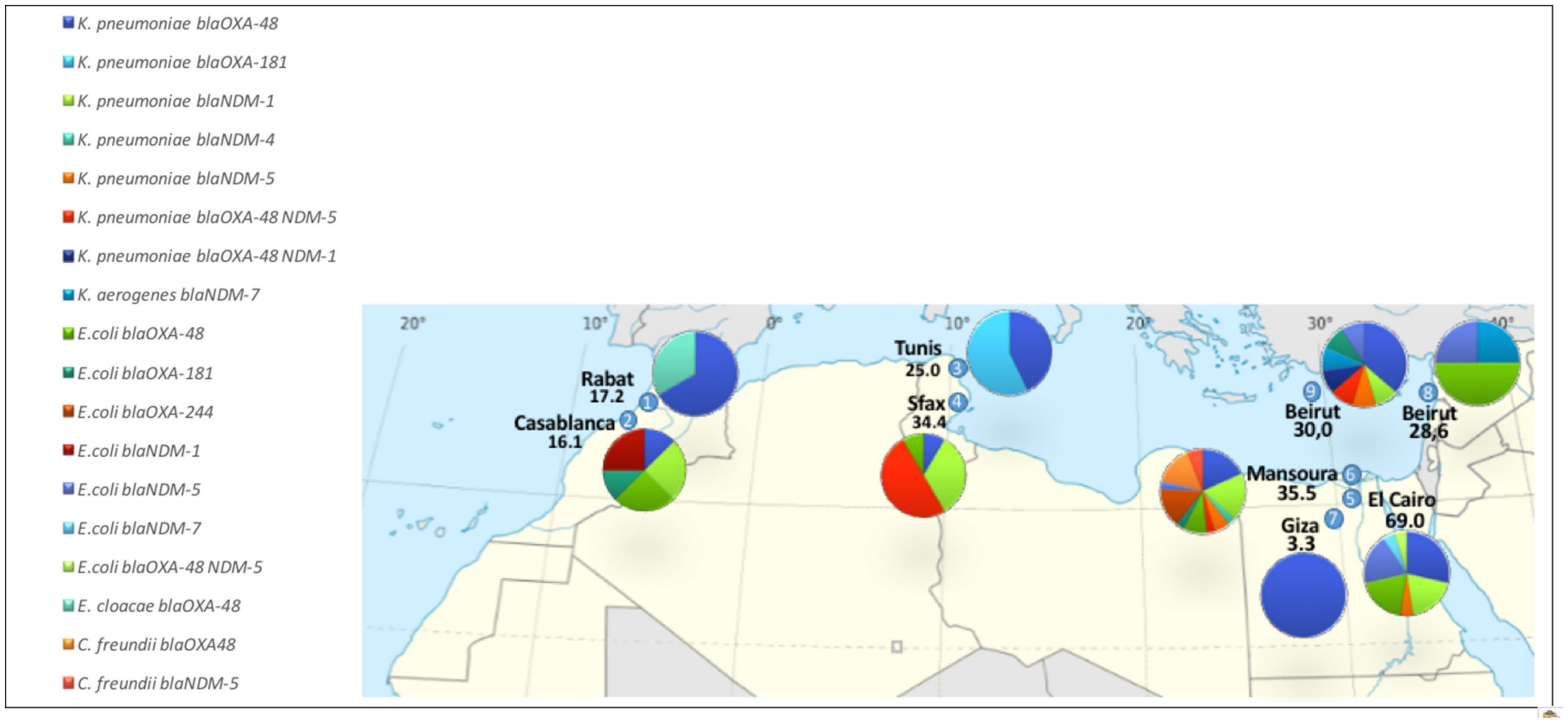
Prevalence of CPE carriage and distribution of the 103 CPE strains, stratified across participating centers.

CPE strains were detected in 73 out of the 256 clinical samples, accounting for 28.5% of the total (Supplementary Table 1). The number of CPE strains varied among carriers, with the majority of carriers exhibiting a single CPE strain (50 cases, 68.5%). The prevalence of CPE carriage ranged from 3.3 to 69.0% across the participating centers (Figure 1).

CPE carriers experienced a prolonged duration of hospitalization before CPE screening. Specifically, 42.5% of CPE carriers had a hospitalization period exceeding 7 days, in contrast to 16.5% of non-carriers (*p* < 0.001). However, antibiotic therapy or hospitalization, both prevalent in the studied population (31.6 and 39.4%, respectively), did not show significant differences based on CPE carriage status.

### 4.3 Detection of colistin-resistant CPE strains

Among the 110 *Enterobacterales* strains cultured on SuperCarba plates, 14 showed resistance to colistin, comprising four *E. coli* and ten *K. pneumoniae.* These 14 strains consisted of 13 CPE strains and one ESBL-producing *E. coli*. Notably, one individual from center C5 was found to carry two of these strains. This resulted in a colistin-resistant CPE carriage rate of 4.7%, affecting three of the eight ICUs (centers C3, C5, and C6). Among the 14 colistin-resistant strains identified, the four *E. coli* strains carried the *mcr1* gene (28.6%)—three of which were CPE strains, alongside the ESBL-producing strain (Table 2). The three CPE strains carrying *mcr1* were isolated from patients in center C6, co-harboring *bla*_OXA-48_, and remained susceptible to amikacin, fosfomycin, and tigecycline. Additionally, the ten colistin-resistant *K. pneumoniae* strains were isolated from eight patients in center C5 and two in center C3. These strains predominantly carried *bla*_OXA-48_ or *bla*_NDM-5_ and exhibited susceptibility solely to tigecycline (100.0%) and amikacin (30.0%).

### 4.4 Strain selection

Epidemiological typing of all strains was performed to select meticulously a representative set of CPE strains. RAPD typing revealed 15 clusters of strains, exhibiting similar RAPD-types in six of the nine centers (Supplementary Table 2). The number of clusters varied from zero (centers C2, C7, and C8) to six (center C6), with a median of one cluster. The clusters consisted of two to six strains each, predominantly represented by *K. pneumoniae* strains. There were ten *K. pneumoniae* clusters, encompassing strains belonging to eight different sequence-types (STs). Notably, the three *E. coli* strains carrying *mcr1*, isolated from patients in center C6, exhibited identical RAPD-types. There was no discernible correlation between a high prevalence of CPE carriers and the number of clusters. For instance, in center C6, where 35.5% of the patients were identified as CPE carriers, six clusters of strains with similar RAPD-types were identified. In contrast, in center C5, which had the highest rate of CPE carriage (69.0% of ICU patients), RAPD-typing revealed only two strains sharing the same RAPD-types among 26 CPE strains.

### 4.5 Whole-genome sequencing

**Figure 2:**
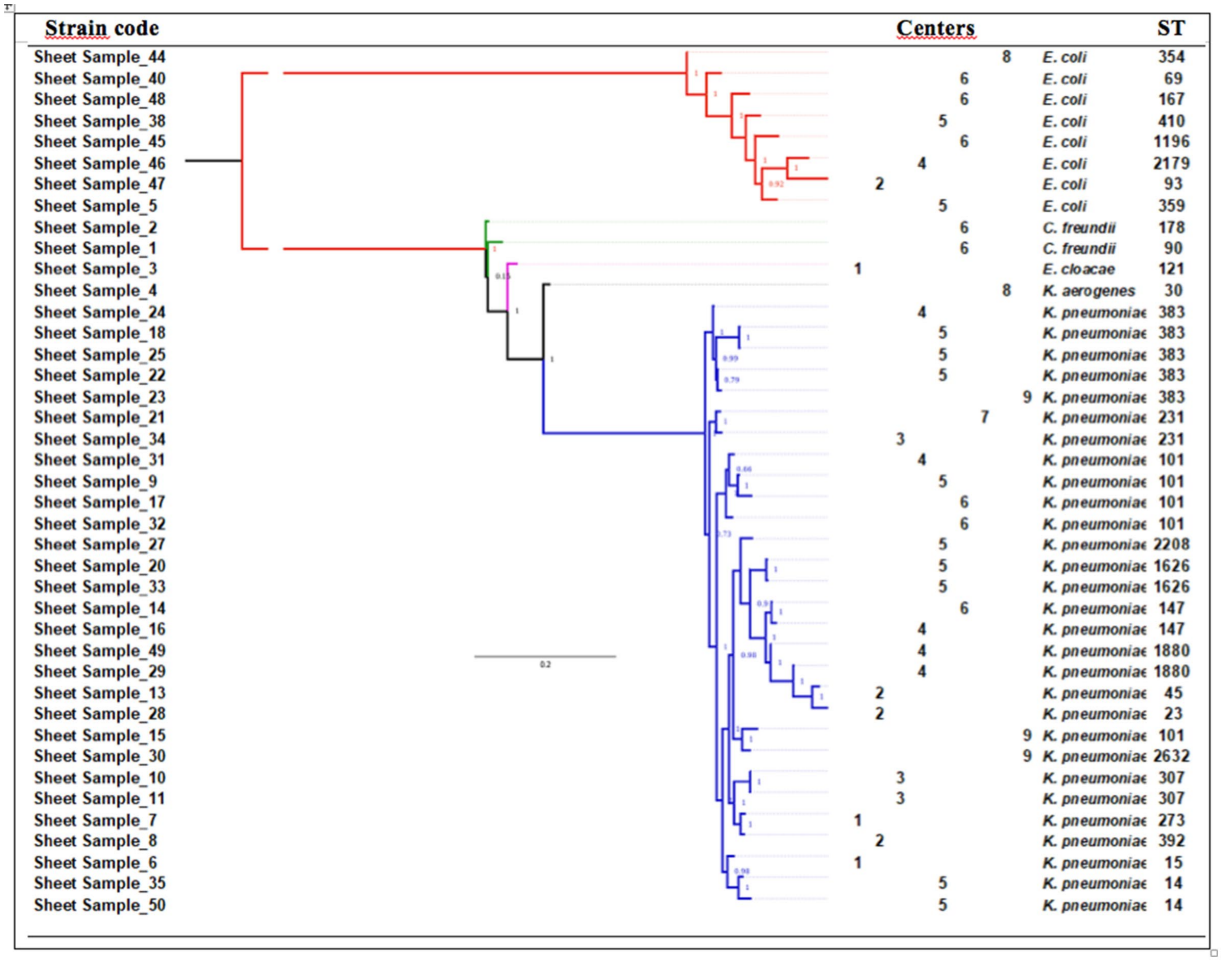
Genetic diversity of the 41 strains studied.

WGS was performed on a set of 40 CPE strains, along with the ESBL-producing *E. coli* carrying *mcr1*. The phylogenetic relationships among the 41 studied strains are depicted in Figure 2. Genetic diversity was notably observed among the eight studied *E. coli* strains, encompassing eight distinct sequence types (STs). Of these, the three CPE strains carrying *mcr1* belonged to ST1196, distinct from the ESBL-producing strain carrying *mcr1*, which was identified as ST359. The 29 *K. pneumoniae* strains exhibited slightly less diversity, comprising 15 different sequence types (STs). The CPE strains identified in the nine ICUs predominantly belonged to various clones. Specifically, only four *K. pneumoniae* clones were found in more than one center: the ST101 clone, detected in centers C4, C5, C6, and C9; the ST383 clone, identified in centers C4, C5, and C9; and the ST147 and ST231 clones, each detected in two centers (centers C4 and C6, and centers C3 and C7, respectively). The five colistin-resistant *K. pneumoniae* strains that underwent WGS were associated with STs 383, 307, and 1626. A detailed analysis of amino acid sequences in these strains revealed a 663-bp sequence in the *K. pneumoniae* Sample_25 genome, replacing the *mgrB* gene with an insertion sequence from the IS1 family. Moreover, multiple mutations were identified in PmrA and PmrB proteins, including a common mutation (T246A) in PmrB shared among all isolates when compared to the colistin-susceptible MGH57578 isolate (Supplementary Table 3). Importantly, no mutations were found in PhoP and PhoQ proteins, and no significant hits to MCR-1 to MCR-10 were detected in the genomes of these colistin-resistant strains.

The resistome study confirmed that most isolates carried genes associated with resistance to fluoroquinolones (92.5%), aminoglycosides (97.5%), sulfonamides (90.0%), macrolides (50.0%), tetracyclines (70.0%), phenicoles (72.5%), and trimethoprim (90.0%) (Supplementary Tables 4, 5). In addition to the numerous genes associated with antimicrobials used in the human clinics, WGS analysis revealed the frequent carriage of genes associated with multidrug resistance, as well as resistance to bicyclomycin — an antimicrobial agent used in livestock environments —, to the antimalarial agent Fosmidomycin, and to numerous heavy metals (Supplementary Table 6).

The virulome study revealed that the majority of *E. coli* and *K. pneumoniae* CPE strains carried genes encoding adhesins, hemolysins, and siderophores (Supplementary Table 7). Additionnaly, *E. coli* strains exhibited the coiled surface structure curlin, which is involved in inert surface colonization and biofilm formation (*csgA*), along with numerous distinct toxin-antitoxin systems that are well-known for genetic element maintenance, virulence, stress resistance and phage inhibition (Qiu et al., 2022).

The plasmid study identified 219 plasmids within the genomes of the 40 CPE strains. The number of plasmid sequences per bacterial genome ranged from zero to 10, with a median value of six. Plasmid sequences were less prevalent among *E. coli* isolates, *Citrobacter* and *E. cloacae* (median value of one plasmid per genome) compared to the *K. pneumoniae* species (median value of seven plasmids per genome) (*p* = 0.005). Genomic comparison of the plasmidic sequences revealed eight plasmid groups (1–8), with plasmids belonging to groups 4, 6 and 7 carried by 65.5, 65.5 and 75.9% of the *K. pneumoniae* strains, respectively (Supplementary Figure 1).

## 5 Discussion

Our comprehensive multicenter point-prevalence survey investigated CPE carriage among 226 ICU patients in nine hospitals across Morocco, Tunisia, Egypt, and Lebanon, conducted pre-COVID-19 pandemic. To our knowledge, this study is unique in providing comprehensive prevalence data on patient colonization in Mediterranean ICUs, distinguishing it as the most recent investigation of its kind.

Firstly, the study unveiled a noteworthy prevalence of CPE strains in eight of the participating ICU centers, demonstrating CPE carriage rates ranging from 16.1 to 69.0% among ICU patients. Additionally, we observed intestinal colonization by colistin-resistant CPE strains in patients from centers 3, 5, and 6. The heightened prevalence of CPE colonization across nearly all ICUs, with the exception of one, increases the risk of subsequent infections in these vulnerable patients harboring highly resistant isolates. This poses inherent challenges in treatment. While it’s highly probable that the prevalence of CPE and colistin-resistant CPE strains has increased during the pandemic (Pascale et al., 2021), our findings are particularly concerning due to the high mortality associated with the identified clones within intensive care settings. Strengthening or set up infection control measures in these ICUs is essential to mitigate the heightened mortality risk among patients. These findings also emphasize the importance of strictly implementing isolation measures for patients originating from the Mediterranean region when transferred to other healthcare institutions.

Secondly, molecular typing of the identified CPE strains unveiled distinct scenarios across centers. In the two ICUs with more than one CPE carrier and no identified clusters (centers C2 and C8), the predominant CPE strains were mostly *E. coli* strains (8/12; 66.6%). WGS analysis revealed carbapenemase-producing *E. coli* belonging to three international high-risk clones [ST410 (Roer et al., 2018), ST167 (Roer et al., 2018; Garcia-Fernandez et al., 2020), and ST69 (Shawa et al., 2022)], known for causing life-threatening infections, particularly affecting critically ill patients with severe underlying diseases and comorbidities, and five globally recognized clones, previously reported in livestock and food products (Stolle et al., 2013; Aizawa et al., 2014; Guo et al., 2015; Wang et al., 2018; Vasconcelos et al., 2020). It appears plausible that the acquisition of these genetically diverse and unrelated *E. coli* strains occurred in the community setting before patients’ hospitalization. In contrast, in the six remaining centers where clusters were identified, CPE strains were predominantly *K. pneumoniae* strains (32/49; 65.3%). The one-day prevalence study did not afford an investigation into the mechanisms of patient acquisition of CPE strains from these clusters. Longitudinal data tracking CPE colonization over time would offer insights into trends, persistence, and factors influencing acquisition and clearance of CPE strains. However, the WGS analysis showed that the identified *K. pneumoniae* strains were predominantly associated with international high-risk clones known for their propensity to thrive in the ICU bedside environment and initiate outbreaks (Weterings et al., 2015; Gordon et al., 2017; Liu et al., 2018; Naha et al., 2021; da Silva et al., 2022; De Koster et al., 2022; Edward et al., 2022; Shi et al., 2022). Thus, our data tentatively suggest nosocomial acquisition of CPE *K. pneumoniae* by patients in the ICUs where clusters were identified. Further evidence is needed for conclusive confirmation.

Concerning the genetic determinants responsible for carbapenem and colistin resistance, our study corroborates previous findings. In alignment with recent global epidemiology of carbapenemase genes (van Duin and Doi, 2017; Pitout et al., 2019), *bla*_OXA-48_ and *bla*_*N*DM-1_ were the most frequently detected genes among the identified CPE strains. Additionally, consistent with observations in Tunisian patients, the studied colistin-resistant *K. pneumoniae* strains did not show the presence of *mcr*-1 to *mcr*-10 genes (Jaidane et al., 2018).

When comparing carriers and non-carriers, our study did not identify prior hospitalization or antibiotic treatment as significant risk factors for CPE colonization. The limited number of ICU patients and the high rate of hospitalization and antibiotic treatment in our sample may have influenced these results. However, our findings confirmed that ICU stays of 7 days or more significantly increased the risk of CPE colonization [1;2]. Once again, the identification of this risk factor reinforces the hypothesis of a major acquisition of CPE strains in the ICU setting during patient care, potentially exacerbated by the failure to identify and isolate CPE carriers within the participating centers

## 6 Conclusion

Our research conclusively establishes the presence and widespread distribution of CPE strains within Mediterranean hosipitals. Significantly, our study adds value by furnishing data on the prevalence of patient colonization and the genetic characteristics of the detected CPE strains—information notoriously challenging to procure. However, the study including a relatively small sample size of ICU patients, with variable enrollment rates across centers, the generalizability of the findings to broader populations may be limited, and the variability in enrollment rates could introduce biases. Prioritizing the awareness of healthcare professionals regarding the escalating threat of antibiotic resistance is crucial, particularly within ICUs. Replicating this study in additional Mediterranean hospitals would promote the implementation of preventive measures and the adjustment of empirical antibiotic treatments for infected ICU patients, if necessary. Finally, our findings highlight the importance of vigilance concerning patients previously hospitalized in Mediterranean facilities and undergoing medical repatriations, to prevent the introduction of CPE into downstream healthcare facilities.

## Data availability statement

The original contributions presented in the study are publicly available. This data can be found here: https://www.ebi.ac.uk/ena, PRJEB71867.

## Ethics statement

The study was conducted in collaboration with hospital directors, attending physicians, and the infection control pilot team in Tours, France, in adherence to French Healthcare recommendations for infection prevention. Ethical approvals were obtained at eight hospital centers, ensuring anonymization of patient and sample data prior to analysis. Data from center 9 were not included in the study. The study was conducted following a protocol in adherence to French Healthcare recommendations for infection prevention. The studies were conducted in accordance with the local legislation and institutional requirements. The ethics committee/institutional review board also waived the requirement of written informed consent for participation from the participants or the participants' legal guardians/next of kin because prior verbal consent was obtained.

## Author contributions

SS: Methodology, Writing – original draft, Writing – review & editing, Formal analysis, Investigation. SD: Formal analysis, Methodology, Validation, Writing – original draft, Writing – review & editing. AB: Data curation, Investigation, Validation, Writing – original draft, Writing – review & editing. KZ: Data curation, Investigation, Writing – original draft, Writing – review & editing. DG: Data curation, Investigation, Validation, Writing – original draft, Writing – review & editing. RE-M: Data curation, Investigation, Validation, Writing – original draft, Writing – review & editing. SE: Data curation, Investigation, Validation, Writing – original draft, Writing – review & editing. IB: Data curation, Investigation, Validation, Writing – original draft, Writing – review & editing. AH: Data curation, Investigation, Validation, Writing – original draft, Writing – review & editing. RC: Data curation, Investigation, Validation, Writing – original draft, Writing – review & editing. ZD: Data curation, Investigation, Validation, Writing – original draft, Writing – review & editing. LM: Conceptualization, Methodology, Writing – original draft, Writing – review & editing. PF: Formal analysis, Methodology, Software, Validation, Writing – original draft, Writing – review & editing. NM-M: Conceptualization, Formal analysis, Investigation, Methodology, Project administration, Supervision, Validation, Writing – original draft, Writing – review & editing.

## Footnotes

^1^Identified using mlplasmid available at https://sarredondo.shinyapps.io/mlplasmids/.

^2^ https://rast.nmpdr.org/rast.cgi

^3^ http://web.mpiib-berlin.mpg.de

^4^Available at: https://cge.food.dtu.dk/services/MLST/.

^5^ https://www.genomicepidemiology.org/

^6^ https://cge.food.dtu.dk/services/ResFinder/

^7^ https://cge.food.dtu.dk/services/MobileElementFinder/

^8^ https://cge.food.dtu.dk/services/VirulenceFinder/
