# Supplementary material for: Carbapenem- and colistin-resistant Enterobacterales in intensive care unit patients in Mediterranean countries, 2019
Source: Front Microbiol. 2024 Apr 12;15:1370553. doi: 10.3389/fmicb.2024.1370553 (PMC11045966; doi:10.3389/fmicb.2024.1370553)
Supplement: Supplementary file 1 [file Data_Sheet_1.zip › Supplem. table 1.docx]

**Supplementary table 1.** Prevalence of CPE carriage and distribution of the 103 CPE strains according to centers.

|  | Number of patients according to centers | | | | | | | | | | All  patients |
| --- | --- | --- | --- | --- | --- | --- | --- | --- | --- | --- | --- |
|  | C1 | C2 | C3 | C4 | C5 | C6 | C7 | C8 | | C9 |  |
| Total number of patients | 29 | 31 | 28 | 32 | 29 | 31 | 30 | 14 | | 30 | 254 |
|  |  |  |  |  |  |  |  |  | |  |  |
| CPE carriers  (%) | 5 (17.2) | 5 (16.1) | 7 (25.0) | 11 (34.4) | 20 (69.0) | 11 (35.5) | 1  (3.3) | 4  (28.6) | | 9  (30.0) | 73  (28.7) |
|  |  |  |  |  |  |  |  |  | |  |  |
| CPE and colRE carriers (%) |  |  | 2  (7.1) |  | 7 (24.1) | 3  (9.7) |  |  | |  | 12  (4.7) |
|  |  |  |  |  |  |  |  |  | |  |  |
| Number of carriers with one CPE | 4 | 2 | 7 | 10 | 14 | 1 | 1 | | 4 | 7 | 50 |
| Number of carriers with >2  CPE | 1 | 3 |  | 1 | 6 | 10 |  | |  | 2 | 23 |
