# Supplementary material for: Carbapenem- and colistin-resistant Enterobacterales in intensive care unit patients in Mediterranean countries, 2019
Source: Front Microbiol. 2024 Apr 12;15:1370553. doi: 10.3389/fmicb.2024.1370553 (PMC11045966; doi:10.3389/fmicb.2024.1370553)
Supplement: Supplementary file 1 [file Data_Sheet_1.zip › Supplem. table 2.docx]

**Supplementary table 2**. Molecular typing of the 103 CPE strains.

| Center | Patient | Species | | | | | Antibiotic resistance | | | | | | | | | | |  | RAPD-type* |  | | MLST  of the CPE strains  associated with clusters | |
| --- | --- | --- | --- | --- | --- | --- | --- | --- | --- | --- | --- | --- | --- | --- | --- | --- | --- | --- | --- | --- | --- | --- | --- |
|  |  |  | | | | | | | | | | | | | | | | | | | |  |  |
|  |  | *E. coli* | *K. pneumoniae* | *K. aerogenes* | *C. freundii* | *E. cloacae* |  | *blaOXA-48* | *blaOXA-181* | *blaOXA-244* | *blaNDM-1* | *blaNDM-5* | *blaNDM-7* | *blaNDM-4* | MIC olistin | *mcr1* | |  |  | |  | |  |
| 1 | 1-1 |  |  |  |  | *+* |  | + |  |  |  |  |  |  | 0.25 | |  |  | R1-Eclo-1 | |  | ST121 | |
|  | 1-2 |  |  |  |  | *+* |  | + |  |  |  |  |  |  | 0.25 | |  |  | R1- Eclo-1 | |  |  |  |
|  | 1-3 |  | *+* |  |  |  |  | + |  |  |  |  |  |  | 0.5 | |  |  | R1-Kpn-1 | |  | ST15 | |
|  | 1-4 |  | *+* |  |  |  |  | + |  |  |  |  |  |  | 0.25 | |  |  | R1- Kpn-1 | |  |  |  |
|  | 1-5 |  | *+* |  |  |  |  | + |  |  |  |  |  |  | 0.25 | |  |  | R1- Kpn-2 | |  | ST273 | |
|  | 1-2 |  | *+* |  |  |  |  | + |  |  |  |  |  |  | 0.5 | |  |  | R1- Kpn-2 | |  |  |  |
| 2 | 2-1 | *+* |  |  |  |  |  | + |  |  |  |  |  |  | 0.25 | |  |  | R2-Eco-1 | |  |  | |
|  | 2-2 | *+* |  |  |  |  |  | + |  |  |  |  |  |  | 0.5 | |  |  | R2- Eco-2 | |  |  | |
|  | 2-3 | *+* |  |  |  |  |  |  | + |  |  |  |  |  | 0.25 | |  |  | R2- Eco-3 | |  |  | |
|  | 2-4 | *+* |  |  |  |  |  |  |  |  | + |  |  |  | 0.25 | |  |  | R2-Eco-4 | |  |  | |
|  | 2-5 | *+* |  |  |  |  |  |  |  |  | + |  |  |  | 0.5 | |  |  | R2- Eco-5 | |  |  | |
|  | 2-2 |  | *+* |  |  |  |  | + |  |  |  |  |  |  | 0.5 | |  |  | R2-Kpn-1 | | |  | |
|  | 2-4 |  | *+* |  |  |  |  |  |  |  | + |  |  |  | 0.25 | |  |  | R2-Kpn-2 | | |  | |
|  | 2-5 |  | *+* |  |  |  |  |  |  |  | + |  |  |  | 0.25 | |  |  | R2-K.pn-3 | | |  | |
| 3 | 3-1 |  | *+* |  |  |  |  | + |  |  |  |  |  |  | 0.5 | |  |  | R3-Kpn-1 | |  | ST307 | |
|  | 3-2 |  | *+* |  |  |  |  | + |  |  |  |  |  |  | 4 | |  |  | R3-Kpn-1 | |  |  |  |
|  | 3-3 |  | *+* |  |  |  |  | + |  |  |  |  |  |  | 8 | |  |  | R3-Kpn-1 | |  |  |  |
|  | 3-4 |  | *+* |  |  |  |  |  | + |  |  |  |  |  | 0.5 | |  |  | R3-Kpn-2 | |  | ST231 | |
|  | 3-5 |  | *+* |  |  |  |  |  | + |  |  |  |  |  | 0.5 | |  |  | R3-Kpn-2 | |  |  |  |
|  | 3-6 |  | *+* |  |  |  |  |  | + |  |  |  |  |  | 1 | |  |  | R3-Kpn-2 | |  |  |  |
|  | 3-7 |  | *+* |  |  |  |  |  | + |  |  |  |  |  | 0.5 | |  |  | R3-Kpn-2 | |  |  |  |
| 4 | 4-1 | *+* |  |  |  |  |  | + |  |  |  |  |  |  | 1 | |  |  | R4-Eco-1 | |  |  | |
|  | 4-2 |  | *+* |  |  |  |  | + |  |  |  |  |  |  | 0.5 | |  |  | R4-Kpn-1 | | |  | |
|  | 4-3 |  | *+* |  |  |  |  |  |  |  | + |  |  |  | 0.5 | |  |  | R4-Kpn-2 | | |  | |
|  | 4-4 |  | *+* |  |  |  |  |  |  |  | + |  |  |  | 0.5 | |  |  | R4-Kpn-3 | | |  | |
|  | 4-5 |  | *+* |  |  |  |  |  |  | + |  |  |  |  | 0.5 | |  |  | R4-Kpn-4 | | |  | |
|  | 4-6 |  | *+* |  |  |  |  |  |  |  |  | + |  |  | 0.5 | |  |  | R4-Kpn-5 | | |  | |
|  | 4-7 |  | *+* |  |  |  |  | + |  |  |  | + |  |  | 0.25 | |  |  | R4-Kpn-6 | |  | ST383 | |
|  | 4-8 |  | *+* |  |  |  |  | + |  |  |  | + |  |  | 0.5 | |  |  | R4-Kpn-6 | |  |  |  |
|  | 4-9 |  | *+* |  |  |  |  | + |  |  |  | + |  |  | 0.5 | |  |  | R4-Kpn-6 | |  |  |  |
|  | 4-1 |  | *+* |  |  |  |  | + |  |  |  | + |  |  | 0.5 | |  |  | R4-Kpn-6 | |  |  |  |
|  | 4-10 |  | *+* |  |  |  |  | + |  |  |  | + |  |  | 0.5 | |  |  | R4-Kpn-6 | |  |  |  |
|  | 4-11 |  | *+* |  |  |  |  | + |  |  |  | + |  |  | 0.5 | |  |  | R4-Kpn-6 | |  |  |  |
| 5 | 5-2 | *+* |  |  |  |  |  | + |  |  |  |  |  |  | 0.5 | |  |  | R5-Eco-2 |  | |  | |
|  | 5-3 | *+* |  |  |  |  |  | + |  |  |  |  |  |  | 0.25 | |  |  | R5-Eco-3 |  | |  | |
|  | 5-4 | *+* |  |  |  |  |  | + |  |  |  |  |  |  | 0.25 | |  |  | R5-Eco-4 |  | |  | |
|  | 5-5 | *+* |  |  |  |  |  |  | + |  |  |  |  |  | 0.25 | |  |  | R5-Eco-5 |  | |  | |
|  | 5-6 | *+* |  |  |  |  |  |  |  | + |  |  |  |  | 0.25 | |  |  | R5-Eco-6 |  | |  | |
|  | 5-7 | *+* |  |  |  |  |  |  |  | + |  |  |  |  | 0.25 | |  |  | R5-Eco-7 |  | |  | |
|  | 5-8 | *+* |  |  |  |  |  |  |  | + |  |  |  |  | 0.25 | |  |  | R5-Eco-8 |  | |  | |
|  | 5-9 | *+* |  |  |  |  |  |  |  | + |  |  |  |  | 0.25 | |  |  | R5-Eco-9 |  | |  | |
|  | 5-10 | *+* |  |  |  |  |  |  |  | + |  |  |  |  | 0.25 | |  |  | R5-Eco-10 |  | |  | |
|  | 5-11 | *+* |  |  |  |  |  |  |  |  |  | + |  |  | 0.5 | |  |  | R5-Eco-11 |  | |  | |
|  | 5-2 |  | *+* |  |  |  |  |  |  |  |  | + |  |  | 16 | |  |  | R5-Kpn-1 | | |  | |
|  | 5-12 |  | *+* |  |  |  |  | + |  |  |  |  |  |  | 0.25 | |  |  | R5-Kpn-2 | |  | ST14 | |
|  | 5-13 |  | *+* |  |  |  |  | + |  |  |  |  |  |  | 0.25 | |  |  | R5-Kpn-2 | |  |  |  |
|  | 5-8 |  | *+* |  |  |  |  | + |  |  |  |  |  |  | 16 | |  |  | R5-Kpn-3 | | |  | |
|  | 5-1 |  | *+* |  |  |  |  | + |  |  |  |  |  |  | 16 | |  |  | R5-Kpn-4 | | |  | |
|  | 5-14 |  | *+* |  |  |  |  | + |  |  |  |  |  |  | 16 | |  |  | R5-Kpn-5 | | |  | |
|  | 5-15 |  | *+* |  |  |  |  | + |  |  |  |  |  |  | 0.25 | |  |  | R5-Kpn-6 | | |  | |
|  | 5-3 |  | *+* |  |  |  |  |  |  |  | + |  |  |  | 0.25 | |  |  | R5-Kpn-7 | | |  | |
|  | 5-16 |  | *+* |  |  |  |  |  |  |  | + |  |  |  | 0.25 | |  |  | R5-Kpn-8 | | |  | |
|  | 5-17 |  | *+* |  |  |  |  |  |  |  | + |  |  |  | 0.5 | |  |  | R5-Kpn-9 | | |  | |
|  | 5-18 |  | *+* |  |  |  |  |  |  |  |  |  | + |  | 16 | |  |  | R5-Kpn-10 | | |  | |
|  | 5-18 |  | *+* |  |  |  |  |  |  |  | + |  |  |  | 0.5 | |  |  | R5-Kpn-11 | | |  | |
|  | 5-19 |  | *+* |  |  |  |  |  |  |  | + |  |  |  | 0.5 | |  |  | R5-Kpn-12 | | |  | |
|  | 5-2 |  | *+* |  |  |  |  |  |  |  |  | + |  |  | 16 | |  |  | R5-Kpn-13 | | |  | |
|  | 5-20 |  | *+* |  |  |  |  |  |  |  |  | + |  |  | 16 | |  |  | R5-Kpn-14 | | |  | |
|  | 5-13 |  | *+* |  |  |  |  | + |  |  |  | + |  |  | 16 | |  |  | R5-Kpn-15 | | |  | |
| 6 | 6-1 |  |  |  | *+* |  |  | + |  |  |  |  |  |  | 0.5 | |  |  | R6-Cfr-1 | |  | ST178 | |
|  | 6-2 |  |  |  | *+* |  |  | + |  |  |  |  |  |  | 0.25 | |  |  | R6-Cfr-1 | |  |  |  |
|  | 6-3 |  |  |  | *+* |  |  | + |  |  |  |  |  |  | 0.25 | |  |  | R6-Cfr-1 | |  |  |  |
|  | 6-4 |  |  |  | *+* |  |  | + |  |  |  |  |  |  | 0.25 | |  |  | R6-Cfr-1 | |  |  |  |
|  | 6-5 |  |  |  | *+* |  |  | + |  |  |  |  |  |  | 0.5 | |  |  | R6-Cfr-1 | |  |  |  |
|  | 6-6 |  |  |  | *+* |  |  |  |  |  |  | + |  |  | 0.5 | |  |  | R6-Cfr-2 | |  | ST90 | |
|  | 6-7 |  |  |  | *+* |  |  |  |  |  |  | + |  |  | 0.5 | |  |  | R6-Cfr-2 | |  |  |  |
|  | 6-8 | *+* |  |  |  |  |  | + |  |  |  |  |  |  | 4 | | + |  | R6-Eco-1 | |  | ST1196 | |
|  | 6-9 | *+* |  |  |  |  |  | + |  |  |  |  |  |  | 4 | | + |  | R6-Eco-1 | |  |  |  |
|  | 6-5 | *+* |  |  |  |  |  | + |  |  |  |  |  |  | 4 | | + |  | R6-Eco-1 | |  |  |  |
|  | 6-5 | *+* |  |  |  |  |  | + |  |  |  |  |  |  | 0.25 | |  |  | R6-Eco-2 | |  | ST69 | |
|  | 6-10 | *+* |  |  |  |  |  |  |  |  |  | + |  |  | 0.25 | |  |  | R6-Eco-2 | |  |  |  |
|  | 6-2 | *+* |  |  |  |  |  |  |  |  |  | + |  |  | 0.25 | |  |  | R6-Eco-2 | |  |  |  |
|  | 6-3 | *+* |  |  |  |  |  |  |  |  |  | + |  |  | 0.25 | |  |  | R6-Eco-2 | |  |  |  |
|  | 6-5 | *+* |  |  |  |  |  |  |  |  |  | + |  |  | 0.25 | |  |  | R6-Eco-2 | |  |  |  |
|  | 6-1 | *+* |  |  |  |  |  | + |  |  |  | + |  |  | 0.25 | |  |  | R6-Eco-3 | |  |  | |
|  | 6-11 | *+* |  |  |  |  |  |  |  |  |  |  | + |  | 0.25 | |  |  | R6-Eco-4 | |  |  | |
|  | 6-11 |  | *+* |  |  |  |  | + |  |  |  |  |  |  | 0.25 | |  |  | R6-Kpn-1 | |  | ST101 | |
|  | 6-10 |  | *+* |  |  |  |  | + |  |  |  |  |  |  | 0.25 | |  |  | R6-Kpn-1 | |  |  |  |
|  | 6-9 |  | *+* |  |  |  |  | + |  |  |  |  |  |  | 0.25 | |  |  | R6-Kpn-1 | |  |  |  |
|  | 6-2 |  | *+* |  |  |  |  | + |  |  |  |  |  |  | 0.25 | |  |  | R6-Kpn-1 | |  |  |  |
|  | 6-3 |  | *+* |  |  |  |  | + |  |  |  |  |  |  | 0.25 | |  |  | R6-Kpn-1 | |  |  |  |
|  | 6-4 |  | *+* |  |  |  |  | + |  |  |  |  |  |  | 0.25 | |  |  | R6-Kpn-1 | |  |  |  |
|  | 6-6 |  | *+* |  |  |  |  |  |  |  | + |  |  |  | 0.25 | |  |  | R6-Kpn-2 | |  | ST147 | |
|  | 6-8 |  | *+* |  |  |  |  |  |  |  | + |  |  |  | 0.25 | |  |  | R6-Kpn-2 | |  |  |  |
|  | 6-5 |  | *+* |  |  |  |  |  |  |  | + |  |  |  | 0.25 | |  |  | R6-Kpn-2 | |  |  |  |
|  | 6-4 |  | *+* |  |  |  |  |  |  |  | + |  |  |  | 0.25 | |  |  | R6-Kpn-3 | | |  | |
|  | 6-1 |  | *+* |  |  |  |  |  |  |  |  | + |  |  | 0.25 | |  |  | R6-Kpn-4 | | |  | |
| 7 | 7-1 |  | *+* |  |  |  |  | + |  |  |  |  |  |  | 2 | |  |  | R7-Kpn-1 | | |  | |
| 8 | 8-1 | *+* |  |  |  |  |  | + |  |  |  |  |  |  | 0.25 | |  |  | R-Eco-1 | | |  | |
|  | 8-2 | *+* |  |  |  |  |  | + |  |  |  |  |  |  | 0.5 | |  |  | R-Eco-2 | | |  | |
|  | 8-3 | *+* |  |  |  |  |  |  |  |  |  | + |  |  | 0.25 | |  |  | R-Eco-3 | | |  | |
|  | 8-4 |  |  | *+* |  |  |  |  |  |  |  |  | + |  | 1 | |  |  | R-Kae-1 | | |  | |
| 9 | 9-1 | *+* |  |  |  |  |  |  | + |  |  |  |  |  | 0.5 | |  |  | R-Eco-1 | | |  | |
|  | 9-2 | *+* |  |  |  |  |  |  |  |  |  | + |  |  | 0.5 | |  |  | R-Eco-2 | | |  | |
|  | 9-3 |  | *+* |  |  |  |  | + |  |  |  |  |  |  | 0.25 | |  |  | R-Kpn-1 | |  | ST147 | |
|  | 9-4 |  | *+* |  |  |  |  | + |  |  |  |  |  |  | 0.25 | |  |  | R-Kpn-1 | |  |  |  |
|  | 9-5 |  | *+* |  |  |  |  | + |  |  |  |  |  |  | 0.25 | |  |  | R-Kpn-2 | | |  | |
|  | 9-6 |  | *+* |  |  |  |  | + |  |  |  | + |  |  | 0.25 | |  |  | R-Kpn-3 | | |  | |
|  | 9-7 |  | *+* |  |  |  |  |  |  |  | + |  |  |  | 0.25 | |  |  | R-Kpn-4 | |  | ST101 | |
|  | 9-8 |  | *+* |  |  |  |  |  |  |  | + |  |  |  | 0.25 | |  |  | R-Kpn-4 | |  |  |  |
|  | 9-1 |  | *+* |  |  |  |  |  |  |  |  | + |  |  | 0.5 | |  |  | R-Kpn-5 | | |  | |
|  | 9-9 |  | *+* |  |  |  |  | + |  |  |  | + |  |  | 0.25 | |  |  | R-Kpn-6 | | |  | |
|  | 9-9 |  |  | *+* |  |  |  |  |  |  |  |  | + |  | 0.25 | |  |  | R-Kae-1 | | |  | |

Grey shading is used in the table to highlight similar RAPD-types.
