# Supplementary material for: Carbapenem- and colistin-resistant Enterobacterales in intensive care unit patients in Mediterranean countries, 2019
Source: Front Microbiol. 2024 Apr 12;15:1370553. doi: 10.3389/fmicb.2024.1370553 (PMC11045966; doi:10.3389/fmicb.2024.1370553)

**Supplementary table 3.** Analysis of the amino acid sequences of MgrB, PmrA, PmrB, PhoP, and PhoQ in the five colistin-resistant *K. pneumoniae* strains.


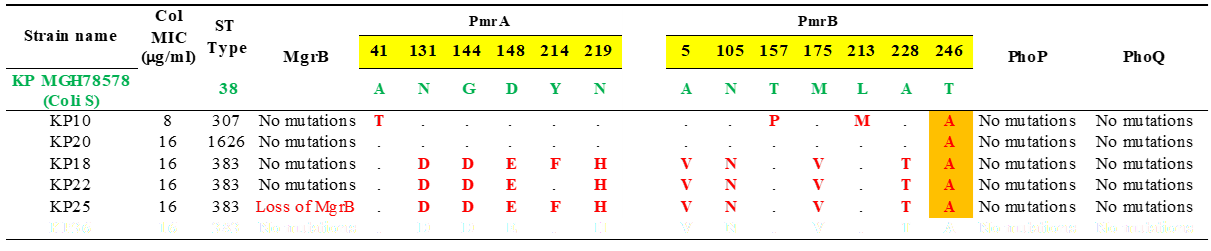

Supplement: Supplementary file 1 [file Data_Sheet_1.zip › Supplem. table 3.docx]
