# Supplementary material for: Carbapenem- and colistin-resistant Enterobacterales in intensive care unit patients in Mediterranean countries, 2019
Source: Front Microbiol. 2024 Apr 12;15:1370553. doi: 10.3389/fmicb.2024.1370553 (PMC11045966; doi:10.3389/fmicb.2024.1370553)
Supplement: Supplementary file 1 [file Data_Sheet_1.zip › Supplem. table 6.docx]

**Supplementary table 6.**

Genes associated with multidrug resistance carried by the 40 CPE strains, and the ESBL-producing *E. coli* carrying *mcr1* (S_5).
