# Supplementary figures and images for: Carbapenem- and colistin-resistant Enterobacterales in intensive care unit patients in Mediterranean countries, 2019
Source: Front Microbiol. 2024 Apr 12;15:1370553. doi: 10.3389/fmicb.2024.1370553 (PMC11045966; doi:10.3389/fmicb.2024.1370553)

**Supplementary figure 1.** Genomic comparison of the 219 plasmids carried by the 40 CPE strains.


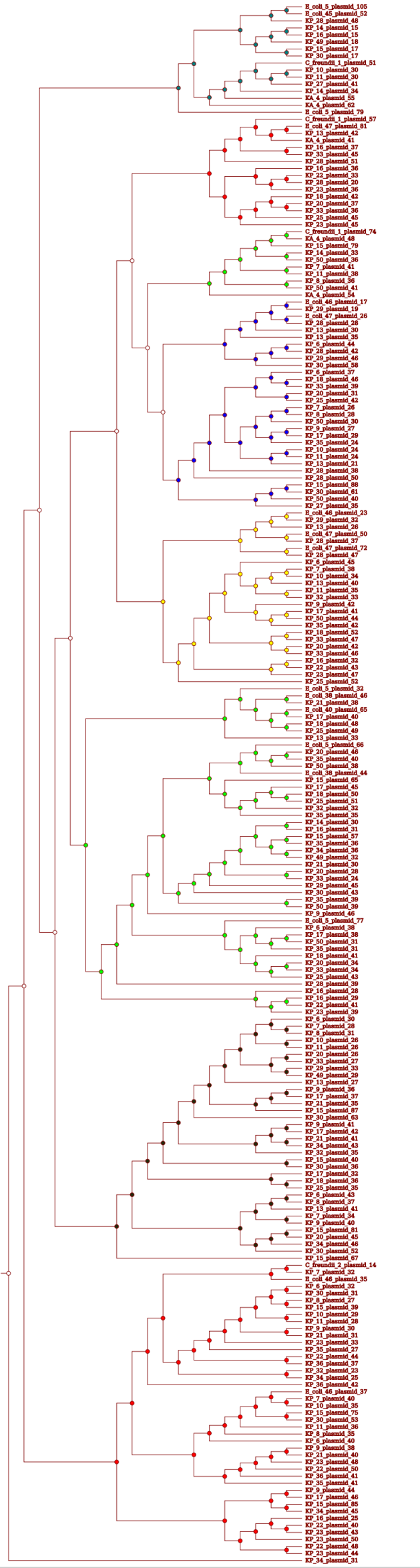

Supplement: Supplementary file 1 [file Data_Sheet_1.zip › Supplem. figure 1.docx]
